# Development and feasibility testing of an artificially intelligent chatbot to answer immunization-related queries of caregivers in Pakistan: A mixed-methods study

**DOI:** 10.1016/j.ijmedinf.2023.105288

**Published:** 2024-01

**Authors:** Danya Arif Siddiqi, Fatima Miraj, Humdiya Raza, Owais Ahmed Hussain, Mehr Munir, Vijay Kumar Dharma, Mubarak Taighoon Shah, Ali Habib, Subhash Chandir

**Affiliations:** a IRD Global, The Great Room, Level 10, One George Street, 049145, Singapore; b IRD Pakistan, 4th Floor Woodcraft Building, Korangi Creek, Karachi 75190, Pakistan; c Interactive Health Solutions, 503, Ibrahim Trade Tower, Shahrah-e-Faisal, Karachi, Pakistan

**Keywords:** Chatbot, Childhood immunization, Artificial intelligence, Natural language processing

## Abstract

**Background:**

Gaps in information access impede immunization uptake, especially in low-resource settings where cutting-edge and innovative digital interventions are limited given the digital inequity. Our objective was to develop an Artificially Intelligent (AI) chatbot to respond to caregiver’s immunization-related queries in Pakistan and investigate its feasibility and acceptability in a low-resource, low-literacy setting.

**Methods:**

We developed Bablibot (Babybot), a local language immunization chatbot, using Natural Language Processing (NLP) and Machine Learning (ML) technologies with Human in the Loop feature. We evaluated the bot through a sequential mixed-methods study. We enrolled caregivers visiting the 12 selected immunization centers for routine childhood vaccines. Additional caregivers were reached through targeted text message communication. We assessed Bablibot’s feasibility and acceptability by tracking user engagement and technological metrics, and through thematic analysis of in-depth interviews with 20 caregivers.

**Findings:**

Between March 9, 2020, and April 15, 2021, 2,202 caregivers were enrolled in the study, of which, 677 (30.7%) interacted with Bablibot (users). Bablibot responded to 1,877 messages through 874 conversations. Conversation topics included vaccination due dates (32.4%; 283/874), side-effect management (15.7%;137/874), or delaying vaccination due to child’s illness or COVID-lockdown (16.8%;147/874). Over 90% (277/307) of responses to text-based exit surveys indicated satisfaction with Bablibot. Qualitative analysis showed caregivers appreciated Bablibot’s usefulness and provided feedback for further improvement of the system.

**Conclusion:**

Our results demonstrate the feasibility and acceptability of local-language NLP chatbots in providing real-time immunization information in low-resource settings. Text-based chatbots can minimize the workload on helpline operators, in addition to instantaneously resolving caregiver queries that otherwise lead to delay or default.

## Highlights

• We assess feasibility of a vaccines chatbot in a low-resource, low-literacy LMIC.
• Despite digital inequities, chatbots are feasible and acceptable in an LMIC.
• Chatbots can be tailored to LMICs by training on local language datasets.
• Caregivers mainly inquired about vaccination schedule and side effects from the bot.
• Integration with digital health registries allows provision of personalized information.

## Acronyms

AI: Artificial Intelligence
BCG: Bacille Calmette Guerin
ChatGPT: Chat Generative Pre-Trained Transformer
EIRs: Electronic Immunization Registries
EPI: Expanded Program on Immunization
HiTL: Human-in-the-Loop
IDIs: In-depth Interviews
IP: Internet Protocol
IQR: Inter-Quartile Range
LMICs: Low-income and Middle-Income Countries
mERA: mHealth Evidence Reporting and Assessment
mHealth: Mobile Health
ML: Machine Learning
NLP: Natural Language Processing
ODK: Open Data Toolkit
OPV: Oral Polio Vaccine
PCV: Pneumococcal Conjugate Vaccine
RASA: Receive Appreciate Summarise Ask
RUBA: Real Urdu Bot Automation
SEIR: Sindh Government’s provincial EIR
SMS: Short Message Service
UNICEF: United Nations International Children's Emergency Fund
WHO: World Health Organization

## 1 Introduction

Despite concerted global focus on childhood immunization, coverage is still not universal and low uptake remains a profound public health challenge with over 19∙7 million children under one year of age missing out on their basic vaccines [1]. Almost 60 % of these missed children reside in ten low-income and middle-income countries (LMICs), including Pakistan [1], where gaps in immunization coverage and timeliness are persistent [2]. Amongst multiple demand and supply side barriers that impede immunization uptake, lack of reliable information and inadequate counselling from healthcare providers cause caregivers' simple questions to remain unresolved, causing them to unnecessarily delay or forgo lifesaving vaccines [3], [4]. Critical gaps in information access cause caregivers to remain deprived of crucial immunization-related information, such as recommended due dates, center timings and location, and side effects [5], [6], [7].

Current information dissemination strategies in Pakistan, such as mass media campaigns [8], paper-based immunization cards [9], [10], and SMS reminders [11], rely on static and unidirectional platforms instead of providing a two-way, conversational experience to caregivers. Mainstream strategies also require high resource allocation and intensify the burden on resource-poor LMIC health systems. Although some existing helplines offer two-way interaction, they necessitate significant human effort and training and do not provide 24/7 functionality [12].

The use of mobile health (mHealth) technologies, such as healthcare chatbots present a viable solution to the interrelated problems of low awareness and absence of interactive communication channels. Chatbots range from simple infobots to Artificial intelligence (AI) based chatbots which provide a two-way personalized experience. AI-based chatbots have demonstrated groundbreaking potential to improve health outcomes in fields such as mental health [13], [14], oncology [15], pediatrics [16], and reproductive health [17], [18], [19]. For instance, Woebot, a mental health chatbot, effectively delivered cognitive therapy to students with symptoms of anxiety and depression [14]. Similarly, Tess, an AI-based behavioral chatbot, provided pediatric obesity and pre-diabetes treatment support to adolescent patients [16]. Despite their popularity in high income countries, health-related chatbots are relatively unknown and underutilized in LMICs where there is dearth of evidence regarding their implementation, feasibility and acceptability, primarily due to the digital inequities driven by low literacy and wealth. Furthermore, immunization chatbots in particular are a relatively unexplored intervention to increase immunization uptake through providing caregivers with actionable information to address trivial immunization-related queries.

We developed Bablibot (Babybot), a local-language, text-based chatbot that connects caregivers to immunization-related information in real-time. Our study investigates the feasibility and acceptability of implementing a local-language immunization chatbot, Bablibot, across a low-resource and low-literacy setting in Karachi, Pakistan.

## 2 Methods

### 2.1 Study design

We deployed a sequential explanatory mixed methods design to measure the acceptability and feasibility of an AI-powered chatbot to serve as a two-way, real-time source of immunization information for Pakistan's low-income communities in Karachi. Our study comprised of three overlapping phases: 1) Development and testing of the chatbot 2) Deployment phase where we invited caregivers visiting the selected immunization centers between March 9, 2020 to August 20, 2020, to interact with Bablibot for their immunization related queries and 3) Post deployment phase between July 27, 2020, and April 15, 2021 where we actively promoted Bablibot through various communication strategies. We simultaneously tracked user and conversation metrics capturing all user-bot interactions, and monitored the technological performance of the chatbot using key metrics such as goal completion rate, accuracy, and fallback rate to ensure optimal performance.

To supplement the quantitative information, we implemented a qualitative component, comprising of 20 phone-based in-depth interviews with randomly selected study participants (with an equal proportion of users and non-users). In depth interviews (IDIs) were conducted to gain insight into user experience, understand why non-users chose not to engage with the bot, and elicit feedback on Bablibot’s utility and acceptability in the community. The interview guide was developed after conducting a review of existing literature and related studies to identify relevant themes that have been covered in existing digital health intervention studies [20], [21], [22], [23], [24]. We evaluated the relevance, quality and appropriateness of the questions identified for inclusion and the study investigators designed the themes to the specific research context and objectives. Specific questions were included to investigate the feasibility of the Chatbot technology. We included questions to understand user's perception of the Chatbot technology (in terms of ease of use, costs) and focused on our outcomes of interest, including accuracy and response time. Given this was a phone-based interview, we were mindful of keeping the questions succinct and included relevant prompts to aid the interviewers in eliciting the desired responses from the participants. We followed the World Health Organization (WHO) mHealth Technical Evidence Review Group’s mHealth evidence reporting and assessment (mERA) checklist to ensure completeness of reporting [25].

### 2.2 Study site

The study was conducted in Korangi and Landhi Towns of Karachi, Pakistan. Mobile penetration in Pakistan is high with almost 78 % of the total population having mobile connections and over 60 million people having access to the internet [26].

Karachi is the largest metropolitan city of Pakistan, with a population of almost 15 million. The population density of Karachi is 24,000 people/km^2^. Immunization services in Karachi are provided through EPI as well as private facilities. There are 360 immunization centers in the city, employing a total of 833 vaccinators. We selected 12 out of 37 immunization centers in Landhi and Korangi towns of Karachi, through convenience sampling. Our selected centers are representative of the overall population. Landhi and Korangi Towns both had an approximate population of around one million at the time of the study. Both towns have an ethnically diverse population comprising a mix of all key ethnic groups of the country: Pashtun, Urdu speaking, Punjabi and Sindhi. The towns are *peri*-urban comprising both urban and rural populations and are home to people ranging from lower-middle-income to low-income communities [27], [28]. We selected 32.4 % (12/37) of the immunization centers which were evenly distributed (as opposed to being clustered) in the selected towns, making them more representative of the study population. The districts within which the two towns are located had a full immunization coverage of 51.8 % and 56.3 %, compared to a national full immunization coverage of 66 % [29], [30].

### 2.3 Study population

The study enrolled participants through two different mechanisms; immunization center-based enrolment as well as recruitment of participants through other communication strategies (SMS reminders, social media campaign, flyers/brochures). Inclusion criteria for immunization center-based enrolment included caregivers visiting any of the 12 selected immunization centers in Korangi and Landhi, child being accompanied by the primary caregiver (mother/father), caregivers owning a mobile phone, and having previously used SMS as a mode of communication. Caregivers of children who were older than 2 years, or who were visiting for the second dose of Measles (last vaccine in the EPI routine immunization schedule) were excluded from the study since they were no longer in the EPI target population and would have completed all their recommended vaccine doses. The inclusion/exclusion criteria for participants enrolled through the other communication strategies could not be verified directly. We assumed that since the people messaged Bablibot directly, they would be within the EPI target age and would have some follow-up vaccination visits left. Additionally, since they messaged directly, it fulfilled the inclusion criteria of owning a phone and having used SMS as a mode of communication previously.

Verbal consent was obtained from caregivers of all eligible children before immunization center-based enrollment. For the participants enrolled through other communication strategies, no formal consent was taken as they had reached out to participate in the study themselves.

All participants who met the inclusion criteria were included in the study, and only those who actively interacted with Bablibot (sent at least one message) were classified as ‘users’.

The study was approved by the Institutional Review Board of Interactive Research & Development (IRD_IRB_2019_11_002).

### 2.4 Vaccination schedule

Pakistan’s routine immunization schedule, in 2019, included BCG (Bacille Calmette-Guérin) vaccine at birth, two doses of Rotavirus at 6 and 10 weeks, three doses of pentavalent vaccine, three doses of pneumococcal conjugate vaccine (PCV) and three doses of oral polio vaccine at 6, 10 and/or 14 weeks of age, typhoid at 9 months of age, and two doses of Measles vaccine at 9 and 15 months of age.

### 2.5 Chatbot development

Bablibot is an artificially intelligent, text-based chatbot, which was developed using Python, the Django Framework. It can operate as a standalone system, but can also be integrated with Electronic Immunization Registries (EIRs). Leveraging Machine Learning (ML) and Natural Language Processing (NLP), with Human in the Loop (HiTL) features, Bablibot converses with caregivers in unconstrained natural language, and in Roman Urdu (Urdu written in English script). Unlike conventional chatbots that respond through rule-based actions based on keywords, Bablibot tries to replicate human conversations, and deploys machine learning (ML) algorithms to continuously self-improve.. The ML model ensures that as the dataset grows, Bablibot becomes increasingly sophisticated and autonomous, improving in terms of accuracy (ability to respond correctly) and breadth (ability to handle a broad range of queries) over time. The HiTL feature allows the bot to default to the human responder when required. It entails a human responder who monitors the bot responses using a web-based dashboard interface. Messages that are not responded to by the bot ware highlighted in bold, indicating that a human responder needs to address them. The messages/conversations are made accessible to all human responders (in the study) to prevent any messages from being missed in case any one responder is unavailable. This allows for prompt and effective handling of any queries that the bot is not able to address, thus ensuring a high-quality user experience. Since March 2020, Bablibot was available via SMS only, but in November 2020, a Whatsapp feature was added in response to community feedback. Bablibot does not necessitate internet or smartphone access and poses no additional cost to users, other than the regular charges of their existing SMS or internet packages.

As part of the bot development, it was initially trained on a transcribed call log of a provincial EIR helpline, which is the only dataset we had available that could mimic the kind of queries we expected the bot to receive post-deployment. However, despite leveraging the EIR helpline data, we did not have enough records to adequately train the ML algorithms. Scarcity of data posed multiple technical and developmental challenges impacting the bot’s ability to respond accurately and making it more dependent on the human responder. We modified certain features such as restricting Bablibot to respond to selected conversation categories and directing the rest to HiTL to address the challenge of a lack of training data for certain queries. Further information on the data preparation and management of Bablibot can be found in the supplementary section.

Leveraging its interoperability with EIRs, we integrated Bablibot with Sindh Government’s provincial EIR (SEIR [26]) after completing development and testing of the bot’s core features. As of April 2021, the Sindh EIR contains data of over 4.6 million children, and provides the bot access to longitudinal, individual-level records for each enrolled child (bio-data and vaccine dates/status), enabling it to provide personalized recommendations based on the child’s age and history. Additionally, due to its ability to retrieve demographic and location data from SEIR, the bot can also provide a list of the nearest immunization centers based on the child’s registered address. However, Bablibot’s ability to engage caregivers is not dependent on EIR data, and it continues to provide guidance and counselling on immunization-related queries to caregivers who are not enrolled in the EIR, or are messaging from a number not linked to their record in the EIR. All responses generated by Bablibot are based on EPI protocols and practices. To ensure the clinical validity of the responses sent via Bablibot, the information was reviewed by public health physicians, vaccine experts and epidemiologists in the study team who ensured that appropriate responses were shared with caregivers. The same team also helped design the immunization decision support system embedded in the Sindh Government’s provincial EIR that used EPI guidelines to schedule current and future vaccines to comply with the WHO-recommended vaccination schedules.

Bablibot is linked to a web-based portal that displays all conversations the bot engaged in and highlights ongoing conversations that require human intervention. Conversation logs can also be downloaded for detailed analysis. The dashboard displays key metrics to monitor bot performance. The web-based dashboard can only be accessed by designated members of the study team each of whom has their unique login credentials.

We collected, preprocessed, and trained Bablibot’s data on a Linux-based server hosted on a private, local cloud service provider of Pakistan - Rapid Compute. To protect against security breaches, we hosted Bablibot on a server allocated for chatbot infrastructure and services within the geographical boundaries of Pakistan, encrypted the database, and allowed for messages to be delivered to the caregiver only if they were received from the server’s IP. We complied with the cloud data hosting protocols in place by the Government and used the same cloud hosting mechanisms currently in use to store Government immunization data.

### 2.6 Study procedures and data collection

The study staff (consisting of study field officers) visited the selected 12 EPI centers in Landhi and Korangi towns, on a rotational basis between March 9, 2020, to August 20, 2020 to enroll caregivers into the study. The study staff were allocated a center for each working day, which they visited once or twice a week. They tried visiting centers on days when the BCG vaccine was being administered (since caregiver volume is highest on these days) to maximize exposure of bot to the target audience.

Caregivers visiting the center with their child(ren) were approached and informed about Bablibot. If the caregiver met the eligibility criteria and provided consent to participate in the study, the study staff collected their basic demographic information, recorded their phone use patterns using electronic forms developed using Open Data Toolkit (ODK), and guided them on how to engage with the bot. Caregivers received monthly reminders highlighting the importance of immunization and reiterating Bablibot’s role as an information source.

In addition to center-based enrolment, the study team promoted Bablibot by sending promotional messages to caregivers registered in the EIR in Karachi, since they had given consent for immunization reminder messages while being enrolled in the EIR. Flyers describing Bablibot were also circulated among caregivers visiting selected EPI centers in Korangi and Landhi towns. User-centric features of Bablibot were highlighted both textually and pictorially, and guidelines on how to message the bot were included. Moreover, we prepared and ran a short Facebook communication campaign for promoting Bablibot from September 15–21, 2021. The content for all activities was developed after incorporating community feedback.

The enrolled study participants who did not interact with Bablibot were not recontacted. Since this was a feasibility study, investigating the acceptability and response rate of the participants to the chatbot, we only introduced Bablibot to the population one time, either through enrollment at study immunization clinics or reaching out through communication strategies and then organically observed how people interacted with the chatbot.

For the qualitative component, we conducted 20 in-depth interviews with a randomly selected subsample of study participants. The interview questionnaire was designed after reviewing the literature, and we modelled the questions on existing studies on AI bots to ensure our questionnaire covered relevant aspects of technology acceptance. Our questionnaire was tailored to the specific context and research questions of the study, taking into consideration the unique challenges and constraints of the LMIC setting in which Bablibot was deployed. Specific questions were included to investigate the feasibility of the Chatbot technology. We included questions to understand user's perception of the Chatbot technology (in terms of ease of use, costs) and focused on our outcomes of interest, including accuracy and response time. In light of the COVID-19 situation, interviews were conducted over the phone. Study participants were stratified by user status prior to selection; those who messaged the bot at least once were considered users, while those who never messaged the bot despite being informed about it during in-person enrollment were considered non-users. Ten consenting participants were interviewed from each stratum. Our objective was to collect detailed feedback on the user experience of caregivers who interacted with Bablibot, solicit suggestions for improvement, explore attitudes towards artificial intelligence (chatbots in particular), and understand non-users’ reasons for not interacting.

### 2.7 Outcome measures

Our primary outcome was to evaluate the feasibility and acceptability of Bablibot through quantitative user and message metrics. User metrics included number of engaged users (people who had messaged at least once), user disconnections (user stopped responding despite ongoing conversation), and user satisfaction. Message metrics were number of messages, number of chat sessions, and range of conversation topics. We supplemented the results with qualitative data generated through in-depth interviews.

Our secondary outcome was to measure Bablibot’s technological performance through technology metrics including interoperability, response speed, goal completion rate (GCR; bot’s ability to provide relevant information: defined as conversations handled correctly by the bot as a proportion of total conversations handled by the bot and not transferred to the human responder), and fallback rate (bot’s dependence on human responder: defined as the proportion of total incoming conversations transferred to the human responder at the first message). As part of validating the model, we also measured Bablibot’s accuracy (percentage of total correct responses), trust score, precision (positive predictive value), recall (sensitivity) and F1 score (harmonic mean of precision and recall). Trust score is a custom measurement calculated as: F1 score*exp (support/n) where support is the number of examples for a specific label, n is the total size of the training set, exp is the exponential function. The trust score gives more weight to labels that have a higher support relative to the overall training set size. By multiplying F1 score by exp (support / n), the metric aims to give more significance to the labels that have a substantial number of examples (high support) compared to the total dataset size (n). The exponential function is used to rapidly amplify values as the input grows.

### 2.8 Statistical analysis

#### 2.8.1 Quantitative analysis

For descriptive analysis, we used frequencies (%) for categorical variables (number of users, number of conversations, gender, occupation etc.), and mean and standard deviation (SD) for continuous variables (household income, enrollment age, average cost of SMS etc.). We compared the socio-demographic characteristics of the two groups to find any significant differences which may have impacted the user’s decision to interact with Bablibot. All analysis was performed using Stata, release 14 (StataCorp, College Station, TX).

#### 2.8.2 Qualitative analysis

All conversations between users and chatbot were recorded and to assess the accuracy of qualitative responses sent by the bot and the completeness of conversations, we categorized each response as ‘accurate’ or ‘inaccurate’ and ‘complete’ or ‘incomplete’ and calculated their proportions from the total responses sent. In-depth interviews (IDIs) conducted on the phone were recorded by the interviewers. Interview recordings were transcribed in Urdu and translated into English for qualitative analysis by research team members who were fluent in both Urdu and English. Transcriptions were further reviewed by a third team member to ensure accuracy and prevent loss of meaning. This approach was taken to maintain the fidelity of the translation, particularly for capturing nuanced and culturally specific phrases and language. IDIs were transcribed verbatim and analyzed by thematic analysis using NVivo software, version 14.2. The investigators first examined the transcripts repeatedly to familiarize themselves with the data and added notes to the transcripts to interpret the data and begin the initial coding process. After this, a coding frame was developed using an open coding approach by enlisting the notes into categories and subcategories, (level 1 (root) and level 2 (sub) codes, respectively). As additional data was analyzed, it was either collated under existing categories or additional categories were added to reflect emerging findings in the codebook. The key themes included 'usefulness of the Chatbot' sub-divided into 'experience of users while interacting with the Chatbot' and 'suggested areas for improvement“. The second key theme was 'Acceptance of AI and Chabot technology', subdivided into 'Understanding of AI technology' and 'Acceptance of AI technology'.

## 3 Results

**Fig. 1:**
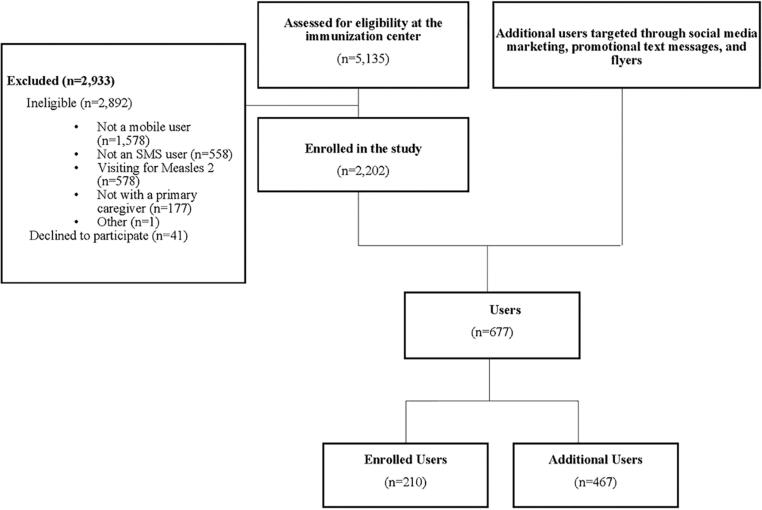
Study participant flow from March 9, 2020 to April 15, 2021 for all users accrued through center-based recruitment and additional communication strategies.

Between March 9 to August 20, 2020, 5,135 caregiver-child pairs visiting the selected EPI centers for routine childhood immunization were assessed for study eligibility; of these 2,892 (56∙3%) were excluded as they did not meet our inclusion criteria. Of those eligible, 2,202 were enrolled in the study while 1∙8% (41/2,892) declined to participate (Fig. 1). More than 100,000 promotional text messages were sent out. A total of 677 people interacted with the Bablibot (i.e. sent at least one message). Of the 677 users, 210 were enrolled in the study at immunization centers while the additional 467 users interacted with Bablibot after becoming aware of Bablibot through other communication strategies or word of mouth.

### 3.1 Participant characteristics

Majority of the caregivers were mothers (67∙4%; 1,484/2,202), with a slightly higher proportion of male children (50∙3%; 1,107/2,202) than female children (49∙7%; 1,095/2,202) (Table 1). The median parental education was 10 years, with 99∙6% (2,192/2,202) mothers being housewives and majority (79∙2%; 1,744/2,202) of fathers working in the private sector (such as sales, bank, or office job). When examining differences between users (n = 210) and non-users (n = 1,992), we found that users’ children were significantly younger than non-users’ children on the day of enrollment into the study (median age: 1∙86 months; IQR: 3∙19 versus median age: 2∙83 months; IQR: 7∙62, 95 % CI: −1.53- −0.37; p = 0.001) and the user’s cohort had mothers who were slightly more educated than non-user mothers (median education: 12 years, IQR: 2, vs median education: 10 years, IQR: 2, 95 % CI: 1.91–2.09, p < 0.001).

**Table 1:** Social demographic characteristics of Chatbot users and non-users enrolled from the selected centers in District Korangi, Sindh between March 9 and August 20, 2020.

	**Total (n = 2,202)**		**Chatbot Users (n = 210)**		**Non Chatbot Users (n = 1,992)**		**% Difference**	**95 % CI**		**P Value**
**Information on Child**	**n**	**%**	**n**	**%**	**n**	**%**				
**Females**	1,095	49∙7	110	52∙4	985	49∙4	3∙0	−4∙10	10∙10	0∙41
**Enrollment age (months)**										
0–1	901	40∙9	106	50∙5	795	39∙9	10∙6	3∙50	17∙70	0∙0030
2–3	503	22∙8	51	24∙3	452	22∙7	1∙6	−4∙48	7∙68	0∙60
4–6	205	9∙3	17	8∙1	188	9∙4	−1∙3	−5∙21	2∙61	0∙54
7–12	565	25∙7	35	16∙7	530	26∙6	−9∙9	−15∙30	−4∙50	0∙0018
12 – 24	28	1∙3	1	0∙5	27	1∙4	−0∙9	−1∙98	1∙84	0∙28
**Enrollment Vaccine**										
BCG/ OPV 0	670	30∙4	74	35∙2	596	29∙9	5∙3	−1∙46	12∙06	0∙11
Penta 1/ OPV 1/ PCV 1	399	18∙1	46	21∙9	353	17∙7	4∙2	−1∙64	10∙04	0∙13
Penta 2/ OPV 2/ PCV 2	298	13∙5	30	14∙3	268	13∙4	0∙9	−4∙06	5∙87	0∙72
Penta 3/ OPV 3/ PCV 3	292	13∙3	27	12∙9	265	13∙3	−0∙4	−5∙17	4∙37	0∙87
Measles 1	543	24∙7	33	15∙7	510	25∙6	−9∙9	−15∙18	−4∙62	0∙0015
**Respondent’s relationship with child**										
Mother	1,484	67∙4	129	61∙4	1,355	68∙0	−6∙6	−13∙50	0∙30	0∙052
Father	718	32∙6	81	38∙6	637	32∙0	6∙6	−3∙00	13∙50	0∙052
**Parent’s Education (years)**										
**Father**										
0	98	4∙5	8	3∙8	90	4∙5	−0∙7	−3∙44	2∙04	0∙64
1–5	88	4∙0	5	2∙4	83	4∙2	−1∙8	−4∙04	0∙44	0∙21
6–10	1,062	48∙4	94	45∙0	968	48∙7	−3∙7	−10∙78	3∙38	0∙31
11–12	532	24∙2	62	29∙7	470	23∙6	6∙1	−0∙36	12∙56	0∙050
>12	416	18∙9	40	19∙1	376	18∙9	0∙2	−5∙39	5∙79	0∙94
**Mother**										
0	98	4∙5	7	3∙4	91	4∙6	−1∙2	−3∙82	1∙42	0∙42
1–5	87	4∙0	2	1∙0	85	4∙3	−3∙3	−4∙91	−1∙69	0∙020
6–10	1,086	49∙4	95	45∙4	991	49∙9	−4∙5	−11∙58	2∙58	0∙23
11–12	578	26∙3	57	27∙3	521	26∙2	1∙1	−5∙22	7∙43	0∙73
>12	347	15∙8	48	23∙0	299	15∙0	8∙0	2∙10	13∙90	0∙0025
**Parent’s Occupation**										
**Father**										
Private job	1,744	79∙2	172	81∙9	1,572	78∙9	3∙0	−2∙50	8∙50	0∙31
Skilled Labor	111	5∙0	8	3∙8	103	5∙2	−1∙4	−4∙16	1∙36	0∙38
Government job	109	5∙0	11	5∙2	98	4∙9	0∙3	−2∙84	3∙45	0∙85
Professional	53	2∙4	6	2∙9	47	2∙4	0∙5	−1∙87	2∙87	0∙66
Manual Labour	137	6∙2	9	4∙3	128	6∙4	−2∙1	−5∙05	0∙85	0∙23
Unemployed	22	1∙0	2	1∙0	20	1∙0	0	−1∙41	1∙41	1∙0
Other	26	1∙2	2	1∙0	24	1∙2	−0∙2	−1∙63	1∙23	0∙80
**Mother**										
Housewife	2,192	99∙6	209	99∙5	1,983	99∙6	−0∙1	−1∙09	0∙89	0∙83
Employed	10	0∙4	1	0∙5	9	0∙4	0∙1	−0∙89	1∙09	0∙83
	**Median**	**IQR**	**Median**	**IQR**	**Median**	**IQR**	**Median Difference**			
Enrollment age for child	2∙73	7∙67	1∙86	3∙19	2∙83	7∙62	0∙97	−1∙53	−0∙37	0∙001
Median Father’s educations	10	2	10	2	10	2	0∙0	−0∙95	0∙95	1∙0
Median Mother’s education	10	2	12	2	10	2	2∙0	1∙91	2∙09	<0∙001

Of all study participants, 97∙9% (2,155/2,202) provided their personal number, while the remaining gave their spouse or other family member’s number (Table 2). More than 98.0 % (2,169/2,202) respondents reported themselves to be frequent mobile phone users (used mobile phones multiple times a day), with 89∙6% (1,974/2,202) using text messages several times a day. Text messages and voice calls were equally preferred modes of phone communication. More users had subscribed to an SMS package as compared to non-users (82∙4% vs 74∙7%, 95 % CI: 2.21–13.19, p:0∙014).

**Table 2:** Mobile Usage Information of Chatbot users and non-users enrolled from the selected centers in District Korangi, Sindh between March 9 and August 20, 2020.

	**Total (n = 2,202)**		**Chatbot Users (n = 210)**		**Non Chatbot Users (n = 1,992)**		**% Difference**	**95 % CI**		**P value**
	**n**	**%**	**n**	**%**	**n**	**%**				
**Personal number given (Yes)**	2,155	97∙9	209	99∙5	1,946	97∙7	1∙8	0∙64	2∙96	0∙086
**Duration of mobile ownership**										
>1 years	2,178	98∙9	208	99∙0	1,970	98∙9	0∙1	−1∙32	1∙52	0∙89
**Frequency of using a mobile phone**										
Several times a day	2,169	98∙5	206	98∙1	1,963	98∙5	−0∙4	−2∙32	1∙52	0∙65
Several times a week	26	1∙2	4	1∙9	22	1∙1	0∙8	−1∙10	2∙70	0∙31
Several times a month	2	0∙1	–	–	2	0∙1	–	–	–	–
Rarely	5	0∙2	–	–	5	0∙2	–	–	–	–
**Frequency of using text message**										
Several times a day	1,974	89∙6	185	88∙1	1,789	89∙8	−1∙7	−6∙28	2∙88	0∙44
Several times a week	59	2∙7	7	3∙3	52	2∙6	0∙7	−1∙82	3∙22	0∙55
Several times a month	39	1∙8	1	0∙5	38	1∙9	−1∙4	−2∙52	−0∙27	0∙14
Rarely	119	5∙4	17	8∙1	102	5∙1	3∙0	−0∙81	6∙81	0∙067
Hardly ever	11	0∙5	–	–	11	0∙6	–	–	–	–
**Preferred mode of communication**										
Text messages	254	11∙5	25	11∙9	229	11∙5	0∙4	−4∙20	5∙00	0∙86
Voice calls	16	0∙7	–	–	16	0∙8	–	–	–	–
Both	1,932	87∙7	185	88∙1	1,747	87∙7	0∙4	−4∙21	5∙01	0∙87
**SMS package subscription (Yes)**	1,660	75∙4	173	82∙4	1,487	74∙7	7∙7	2∙21	13∙19	0∙014
**Opens text messages from unknown numbers (Yes)**	18	0∙82	–	–	18	0∙90	–	–	–	–
	**Mean**	**SD**	**Mean**	**SD**	**Mean**	**SD**	**Mean Difference**	**95 % CI**		**P value**
**Average cost of one text message (PKR)**	2∙1	1∙0	2∙0	0∙7	2∙2	1∙0	−0∙2	−0∙02	0∙41	0∙080
**Average cost of package**	343∙9	371∙9	373∙3	280∙9	340∙5	380∙9	32∙8	−104∙9	39∙4	0∙37

### 3.2 Bablibot Uptake, Acceptability, and demand

Between March 9, 2020 and April 15, 2021, Bablibot accrued 677 users, responded to 1,877 messages (excluding 307 exit survey responses), and participated in 874 distinct conversations (data not shown). The conversations took place both on SMS (80 %; 698/874) and Whatsapp (20 %; 176/874).

Caregivers messaged Bablibot for various queries, ranging from vaccine side effects to logistical information about center timings (Table 3). Around one-third of the conversations (32∙4%; 283/874) were about the immunization schedule or the EPI services, with many caregivers asking “*When will my child get his next injection?”* Queries about the immunization schedule or EPI services also included questions regarding number of doses, vaccination cost, EPI polio teams’ outreach activities, and what to do if the child’s EPI card was lost. Questions about delayed or missed immunizations constituted 16∙8% (147/874) of the conversations. Caregivers asked if they could delay their child’s vaccinations by a few days because of the COVID-19 lockdown, illness, weather, or personal commitments. Others inquired if they could get their child vaccinated if the child was a few months late or over the age of two years.

**Table 3:** Types of Conversations Bablibot catered to from March 9, 2020 to April 15, 2021.

**Conversation Category**	**n = 874**	**%**
Vaccination schedule and EPI-related queries	283	32∙4
Delayed or missed vaccination	147	16∙8
Adverse events and post-vaccination discomfort	137	15∙7
Center related information	134	15∙3
Other immunization and non-immunization queries	173	19∙8

Almost 15∙7% (137/874) conversations were initiated by caregivers seeking Bablibot’s guidance regarding side effects following vaccination, such as fever, pain or swelling (for severe side effects and for persistent symptoms caregivers were advised to seek medical attention) (Table 3). Other caregivers messaged Bablibot to preemptively learn about and prepare for potential side effects, even if the child was not experiencing any side effects at the time of the message. Another 15∙3% (134/874) of the conversations were about immunization center information. Specific questions were about center timings (including during lockdown, holidays, and flooding), center location, or the designated days for the Measles or BCG vaccine at the nearest immunization centers (BCG and Measles vaccines are only administered on specific days at EPI immunization centers).

In terms of user satisfaction and acceptability, the interactions between Bablibot and the caregivers yielded a satisfaction rate of 90 %, based on the 307 responses of a short exit survey administered at the end of each chat session. The voluntary exit survey was offered to all Bablibot users. In more than 100 chat sessions, caregivers appreciated the service by expressing gratitude (by using the phrases “thank you” and “this was helpful”) and indicating that caregivers had received the required information. Of the 677 users, 20 % (139/677) interacted with the bot on multiple occasions. In 87 chat sessions, the users stopped responding despite ongoing conversations or did not send their questions despite initiating conversation.

### 3.3 Technological performance

Following development of the beta version in March 2020, Bablibot was fully functional > 95 % of the time, and operated as intended. The dashboard connected to the bot also functioned as intended, enabled continuous monitoring, and prompted bug identification. Since October 2020, Bablibot was fully functional 100 % of the time. The average response time for the bot was within two minutes.

Between March 2020 to April 2021, the cumulative goal completion rate (GCR) was 43 %. After resolving technical challenges, we achieved a cumulative GCR of 61 % from January 2021 to April 2021. Additionally, we observed a fallback rate of 63 % during our study. Between August 2020 and April 2021, Bablibot had an average accuracy of 99.3 %, a trust score of 0.91, precision of 87.2 % and recall of 86.3 %. and an F1 score of 87.3 %.

### 3.4 Qualitative results

A total of 20 (10 users and 10 non-users) study participants participated in the phone-based in-depth interviews. Doctors were reported as the primary source of health-related information for almost all caregivers (90 %; 9/10 users and 100 %; 10/10 non-users), followed by family and/or relatives (10 %; 1/10 users and 20 %; 2/10). Of all users, three (30 %) cited referring to Bablibot for health-related information. Use of digital sources such as the internet was relatively uncommon amongst participants, with only 30 % (3/10) users and 20 % (2/10) non-users using the web for health information. For immunization-specific information sources, users cited Bablibot (60 %; 6/10), EPI cards (20 %; 2/10), EPI centers (20 %; 2/10). Half of non-users reported either not having the need to consult anyone for immunization specific information (50 %; 5/10) or referred to EPI centers (30 %; 3/10), EPI card (20 %; 2/10), or relatives (30 %; 3/10) as the primary information source.

We identified two themes through our qualitative interviews with study participants: 1) usefulness of Bablibot and 2) acceptance of artificial intelligence and chatbot technology.

#### 3.4.1 Usefulness of Bablibot

##### 3.4.1.1 Experience

Majority (8/10) of the users we interviewed shared a positive experience of using Bablibot. Caregivers appreciated Bablibot in terms of being easily accessible and providing accurate and timely information and saving them an unnecessary trip to the immunization center, underscoring the convenience and reliability of the platform. One caregiver responded, “*Bablibot has made my life easier, first I had to go to the center, which was a little far from my home, for trivial questions. Now I can get the information while sitting at my home*” (ID-1953), while another said “*It will be beneficial for people with firstborn children, especially. Children usually get fever after their first vaccine, parents can refer to Bablibot then*” (ID-1912).

Three caregivers, despite being satisfied with the overall service, reported late replies or no reply on their second interaction.

Caregivers reported using Bablibot mainly for questions related to side effects management, timing and due date of vaccines, or the child’s general health. For non-users, the most cited reason for not messaging was *‘no need’* for immunization information (during the study period), mainly because of already having experience with childhood vaccines. Some non-users also hinted that only educated people will be able to use Bablibot, limiting its reach.

##### 3.4.1.2 Impact and areas of improvement

When asked about Bablibot’s impact on immunization uptake, user caregivers commented that Bablibot will increase immunization uptake by increasing awareness and disseminating information quickly, especially for mothers who otherwise have limited access to information. Although unsolicited, two out of ten users reported having informed others in their social circle about Bablibot as well. Non-users, although did not feel the need for using Bablibot during the study period, still believed in the potential of Bablibot to have an impact on immunization uptake by providing remote and immediate access to immunization information.

Most of the caregivers suggested that Bablibot should be accessible in multiple languages so more people can use it. Caregivers would also like the answers to reach them quickly, with reliable, complete, and to the point information. One mother suggested *“… the response should come instantly; one should not have to wait”* (ID-1835).

#### 3.4.2 Acceptance of AI and chatbot technology

##### 3.4.2.1 Understanding of AI technology

Caregivers had limited understanding of AI and chatbot technology. Only 20 % (2/10) users and 10 % (1/10) non-users were aware of what a chatbot was, but none had used it before. For Bablibot specifically, 50 % (5/10) of users and 10 % (1/10) of non-users (even though unaware of the word ‘chatbot’), knew that Bablibot responded through a system i.e. they understood that a computer was responding to their messages.

##### 3.4.2.2 Acceptance of chatbot technology

Regarding chatbot’s acceptability, 90 % (9/10) users said they do not have any concerns with consulting a chatbot for their child’s immunization; however, 40 % (4/10) users commented they would still want to know who was sending the responses. On the other hand, non-users indicated they would be concerned in interacting with a bot in matters pertaining to their child. The Human in the Loop feature was appreciated by almost all users (60 %; 6/10) and non-users (90 %; 9/10), who said human monitoring makes them feel comfortable seeking information from Bablibot.

When questioned regarding the community’s reaction towards Bablibot, 20 % (2/10) of non-users responded that the community would have mixed reactions, with educated people more likely to accept Bablibot, and 20 % (2/10) believed that their community would not be willing to use Bablibot. However, 30 % (3/10) of non-users and 50 % (5/10) users believed that people in their community would accept and react positively towards Bablibot.

## 4 Discussion

Our results suggest that a local language AI-based chatbot is a feasible and acceptable intervention for providing immunization information to caregivers of children in a limited resource and low literacy setting. To our knowledge, our study was the first to evaluate an NLP-based bot in a low-resource setting like Pakistan, and the first to examine acceptability of any kind of health chatbot among an urban-poor population.

At the time of this study, there were limited examples of local-language (Urdu) Chatbots. Two of the Chatbots (a reproductive health awareness chatbot and the Government of Pakistan’s COVID-19 Chatbot) [32] only allowed selecting options from a pre-defined list of queries. Another non-health text-based Chatbot conversed in both English and Urdu and relayed queries to a human responder when it was unable to provide the required information. Unlike Bablibot, none of these Chatbots operated without internet connectivity or displayed context awareness (determined through first-hand experience of interacting with the bots). Since the conclusion of this study, there has been an increase in the number of local-language Chatbots such as an AI-enabled Urdu voice recognition bot named RUBA (Real Urdu Bot Automation) that is capable of performing simple activities such as checking the amount in the user’s bank account and sending text messages through users phone number [34]. Another NLP Urdu conversation agent ‘*Saathi’* [35] was also launched, based on the Python-based machine learning RASA [36] framework for conversational AI. The Chatbot was designed for the elderly, to remind them of their medications, obtain daily news highlights, and interact with their loved ones. A cross-validation of the Chatbot revealed a ratio of misclassifications to correct classifications of 0.247, indicating room for improvement and the need for a fallback policy, similar to Bablibot.

With the advent of Large-Language-Models such as ChatGPT and its potential to be utilized for healthcare, further work is underway to evaluate ChatGPT for facilitating the adaptation of clinical guidelines. A recent study [37] identified multiple recurrent errors, including misreporting and non-reporting errors in adapting and synthesizing clinical guidelines for diabetic ketoacidosis, underscoring the need for expert human intervention and validation. In a similar study [38], ChatGPT’s accuracy, clarity and efficacy scores were 3.7, 3.8 and 3.3 (out of 5) respectively, for questions about symptoms, treatment and diagnostics of gastrointestinal issues. Although we cannot draw a similar comparison on these indicators with Bablibot, these findings also highlight the need for further refinement of the AI-based Chatbot models.

Regarding the acceptability of Bablibot, caregivers showed interest in interacting with Bablibot, with many caregivers approaching Bablibot on repeated occasions. Our findings are in line with previous research demonstrating usability and acceptability of AI-based chatbots for engaging people for health-related information [13], [39] and inducing behavioral change . A study in Brazil [39] found a mother-baby chatbot to be useful in advancing child health, and over 90 % of the 142 participating women were satisfied with using it in terms of usability, content clarity, and quality of information. Other health-related chatbots in the field of mental health,[14] oncology,[15] reproductive health [19] have also demonstrated high potential in terms of providing timely and accurate information and have yielded high end-user acceptability. However, it is worth noting that, unlike Bablibot, no health chatbot is available via SMS or Whatsapp. A more recent study [40] on a diabetes education chatbot reported high overall satisfaction for chat content, length, and frequency, with 87 % of participants reporting increased self-care confidence. Our findings also showed a high (>90 %) satisfaction rate of Bablibot. The high satisfaction rate was in part due to the presence of human monitoring (human-in-the-loop feature) which ensured that users received the information they were seeking, even in cases where the bot was unable to provide an accurate response. The demonstrated feasibility and acceptability of the Bablibot in a low-resource setting is an important finding as the digital inequities between the high- and low-income countries have limited the potential of innovative, cutting-edge innovations in many low-resource settings. Our study shows that despite resource and literacy limitations, health-related chatbots can be implemented in LMICs, with caregivers showing high acceptability and interaction with the platform.

We found Bablibot to be a feasible solution for providing guidance to caregivers by acting as a bi-directional, 24/7 chat service, easily available to them at minimal cost. In Pakistan, other common sources of immunization information are EPI cards, vaccinators present at immunization centers, and call-based helplines. A federal national level helpline has been set up by EPI with support from WHO and UNICEF with around 150 lines and 150 staff members including helpline operators, supervisors, and doctors [41]. The helpline operates from 8:00 am to midnight, seven days a week, and receives over 70,000 calls per month catering to queries regarding immunization, vaccine-preventable diseases, polio, and COVID-19 [41]. A provincial immunization helpline in Sindh is also available for caregivers which received 2,327 calls per month in 2021. [42] The immunization questions received on these helplines were similar to those catered to by Bablibot, however, unlike these phone-based helplines, Bablibot provided immediate, accurate, easily accessible immunization information with 24 h access. Moreover, interoperability with the provincial electronic immunization registry provided Bablibot with the unique advantage of providing personalized information based on the individual child’s immunization history. Personalized, data-derived messages tailored to the child’s individual needs (such as vaccine reminders, child’s vaccination due dates), are more likely to make caregivers receptive to the incoming information as opposed to mass messages, and elicit the required behavioral response for increasing immunization uptake.

NLP-based chatbots, such as Bablibot have the potential to address a country’s health information gaps that impede immunization uptake. Our results demonstrate that caregivers in Pakistan require ongoing guidance throughout their child’s immunization journey for simple yet crucial information such as vaccination dates or side effects. Chatbots, such as Bablibot, have the potential to address these concerns instantaneously, which if left unaddressed, trigger vaccine-hesitant behavior. [43] Moreover, by being a remote and confidential information source, chatbots can address gender gaps in information access in areas where women lack the ability to seek information and health services due to local gender norms [4], [7], [8]. In the post-COVID world, where in-person counseling is difficult, chatbots can play a key role in providing key health information, ensuring health services are minimally disrupted. Lastly, interoperability with electronic health registries enhances the bot’s potential to expand into other areas of public health, including antenatal care, child health, and COVID vaccinations, especially after it has garnered community approval and trust. For Bablibot particularly, integration with Government’s EIR, for example, allows for easy scale up of Bablibot across other areas of Pakistan.

Our study has certain limitations; since our chatbot was the first of its kind immunization chatbot there was limited training data available in Urdu, limiting our ability to develop a full context-aware chatbot. It has been proven that non-english chatbots are bound to face more technological challenges during their development [44]. However, after the initial development and testing of Bablibot as outlined in this study, the constantly evolving dataset consisting of real time caregiver-bot conversations, will allow Bablibot’s ML algorithms to become more sophisticated, improving its ability to respond accurately. As demonstrated by our findings, Bablibot faced a higher fallback rate since our focus was to improve the bot's ability to handle the specific conversations it was trained on, rather than attempting to handle all types of conversations. Moreover, it was operational during the time when bot technologies were not as advanced as they are today, and especially in an LMIC setting where there was limited availability of local data. However, in such circumstances, even being able to handle more than one-third of the conversations is a remarkable accomplishment. Given that our work focused on studying the feasibility of a local-language chatbot, we did not include the measure of technology readiness level (TRL). The TRL is vital for a comprehensive assessment of innovative technological solutions and provides valuable insights into the system's maturity and reliability of technology components during the research, development, and deployment phases, allowing for consistent comparisons and realistic understanding of risks. The relevance of TRL in artificial intelligence has been demonstrated by multiple studies [45], [46] and therefore, we recommend its use in future AI-based chatbot assessments. The participating immunization centers were selected on the basis of convenience sampling, potentially impacting the generalizability of the results. However, by encompassing diverse study site population and inviting all caregivers visiting the selected centers, we aimed to minimize the bias. Additionally, we could not verify the inclusion/exclusion criteria for a sub-sample of participants who were enrolled in the study through mass communication channels. Although we can reasonably assume that these participants fulfilled the inclusion criteria as explained in the methods section, the results should be generalized with caution. Also, due to limited time and the COVID-19 situation, we had a small sample size (20) for our phone based in-depth interviews, restricting our ability to generalize the results to a wider population. Furthermore, due to the novelty of chatbots in Pakistan, we had to spend a relatively large proportion of our resources in marketing our bot and onboarding users; we believe that for some users, low awareness might have contributed to reluctance in accepting and using Bablibot right away. As chatbots become more commonplace in Pakistan, user acceptability and retention is expected to be even higher.

As a way forward, our study shows the potential of incorporating local language AI-based chatbots into the clinical immunization workflow to enhance patient engagement and improve patient education regarding immunization. Integration of such Chatbots with electronic health records could revolutionize the delivery of accurate, personalized information while dispelling misconceptions and misinformation regarding immunizations. Our next steps will be to leverage incoming Bablibot data to train the model further and explore avenues for additional similar training data sets considering the upsurge in local language chatbots post this study. Future avenues for research include training and testing the Chatbot in other settings and with additional caregivers to capture the diversity of queries and strengthen the model further. Any future evaluation of the model would also aim to expand our caregiver sample size to obtain more representative responses on Bablibot feedback. Additionally, a pertinent concern with the use of Chatbots is assessing the possible risk of biases, fairness, and over-reliance which was not part of the scope of this study. These are important aspects that we intend to cover in any future Chatbot evaluations. There are a number of measures we plan to deploy to address these risks, including ensuring a diverse and representative dataset for training the bot, developing guidelines for content moderation and filtering to prevent harmful or biased responses, regularly assessing the chatbot's responses for fairness and equity, employing human reviewers to monitor and evaluate the chatbot's interactions through regular audits as well as seeking end-user feedback, and training the developers and teams working on chatbots about the ethical implications and challenges related to bias, fairness, and over-reliance.

AI-based chatbots are feasible and acceptable for providing real-time immunization-related information to caregivers in a limited resource and low-literacy setting. Chatbots have additional value when linked to EIRs through provision of 24/7 personalized and contextualized information to instantaneously resolve caregiver queries that otherwise lead to delay or default. Conversation data generated through Bablibot also provides learning infrastructure for future health-related bots in South Asia. While our study is a starting point for demonstrating the potential impact of chatbots on immunization, a more rigorous evaluation is warranted to evaluate the full potential of chatbots in raising parental awareness and improving immunization coverage and timeliness.

Summary Table.

What was already known on the topic.

• We searched PubMed and Google Scholar between February 19, 2021 and October 1, 2021, using the search terms “chatbot”, “childhood immunization”, “health’, “artificial intelligence”, and “natural language processing” and found 5,547 articles, out of which we shortlisted 61 that were related to health-related chatbots mainly in the field of mental health, oncology, and reproductive health.
• Evidence of health-related chatbots in low-income and middle-income countries (LMICs) was limited.
• We did not find a single study investigating immunization-based chatbots across low or high-income countries.

What this study added to our knowledge.

• A local-language, text-based chatbot (called Bablibot) is a feasible solution for disseminating accurate, real-time immunization information to caregivers in low-resource settings.
• There was high acceptance and high user engagement with Bablibot.
• The conversation data generated through our study has the potential to provide learning infrastructure for future health-related natural language processing (NLP) bots in South Asia.
• Our findings make the case for LMICs to consider utilizing chatbots to optimize health education and information provision to caregivers to improve immunization uptake.

## CRediT authorship contribution statement

**Danya Arif Siddiqi:** Formal analysis, Funding acquisition, Investigation, Methodology, Supervision, Writing – original draft, Writing – review & editing. **Fatima Miraj:** Project administration, Data curation, Writing – original draft, Formal analysis. **Humdiya Raza:** Software. **Owais Ahmed Hussain:** Software, Supervision. **Mehr Munir:** Methodology, Project implementation, Data curation. **Vijay Kumar Dharma:** Resources, Project implementation. **Mubarak Taighoon Shah:** Resources, Project implementation. **Ali Habib:** Supervision, Funding acquisition, Software. **Subhash Chandir:** Supervision, Conceptualization, Formal analysis, Funding acquisition, Investigation, Methodology, Software, Validation, Visualization, Writing – review & editing.

## Declaration of Competing Interest

The authors declare that they have no known competing financial interests or personal relationships that could have appeared to influence the work reported in this paper.
