# Supplementary material for: Development and feasibility testing of an artificially intelligent chatbot to answer immunization-related queries of caregivers in Pakistan: A mixed-methods study
Source: Int J Med Inform. 2024 Jan;181:105288. doi: 10.1016/j.ijmedinf.2023.105288 (PMC10750258; doi:10.1016/j.ijmedinf.2023.105288)
Supplement: Supplementary data 1 [file mmc1.docx]

**Data Preparation and Management for Chatbot**

The chatbot was trained using DNN (Deep Neural Network) comprising of 100 fully connected layers in Tensorflow^^[[1]](#footnote-1)^^, a Machine learning library based on Python programming language.

**Dataset:**

A base data set, comprising a corpus of 500 SMS messages in Roman Urdu language, was collected directly from mobile devices used by healthcare providers, and loaded into a document-based database (we used MongoDB as database management system). This base data set was manually synthesized. The model was trained on routinely collected immunization data, therefore it was not possible to add additional research variables

The data was then randomly shuffled and split; 50% was used for model training and 50% for validation and evaluation. Standard text mining methods were used to synthesize and transform data.

1. Text transformation:

Each example was first converted into lower case, since Python is a case sensitive language. Leading and trailing spaces were removed. All special characters were replaced with fixed placeholders. For example, new line characters were replaced with “<<newline>>”

1. Non-dictionary terms:

The terms which did not provide any contextual information were also handled. A dictionary of 95% most frequently used names in the Immunization registry (SEIR) was collected from the EPI database, scanned in the data and replaced with a placeholder. Similarly, the data strings in commonly used formats^^[[2]](#footnote-2)^^ were also dealt with the help of regular expressions and replaced with placeholder.

1. Tokenizing:

We used *word tokenizer* function of NLTK^^[[3]](#footnote-3)^^ library to tokenize sentences into words. The tokens were them lemmatized and stemmed with the help of a dictionary specifically created to represent Roman word alternatives. For example, in the corpus, the word “daughter” has aliases “bachi”, “bachee”, “beti”, and “baby girl”. All of these aliases were treated as single term. The team is provided with an interface to update this dictionary as new terms and their alternatives are discovered.

A wide range of data mining techniques such as tokenizing, stemming, and lemmatization were applied using the NLTK (a popular library for text mining) along with the addition of generated synthetic examples to enrich the dataset. The scarcity of data did not leave ample room to discard any examples, therefore missing data was retained in the datasets.

**Training and Evaluation**

From the pre-processed dataset, a BOW (bag of words) model was generated; this is a simple approach which assigns a number to each word for faster processing. The Tensorflow model was supplied with this BOW dataset and trained using the following parameters:

1. Number of epochs: 100
2. Batch size: *10*
3. Hidden NN layers: 100
4. Estimator: Regression model
5. Activation function: Softmax^^[[4]](#footnote-4)^^
6. Optimizer function: Adam^^[[5]](#footnote-5)^^
7. Learning rate: 0.1

The trained model was evaluated on the test data set using 7 metrics:

1. Accuracy: (TP+TN) / (TP+TN+FP+FN)
2. Error rate: (FP+FN) / (TP+TN+FP+FN)
3. Recall: TP/(FP+FN)
4. Precision: TP/(TP+FP)
5. F1 Score: Harmonic mean between Precision and Recall
6. Trust score: Custom evaluation function, calculated as the product of F1 score with the Exponent of ratio between TP and Total examples, i.e., $f1 score \times expexp \left( \left( TP+1 \right)/\left( N+1 \right) \right)$

The Trust score gives more weight to labels that have a higher support relative to the overall training set size. By multiplying F1 score by exp(support / n), the metric aims to give more significance to the labels that have a substantial number of examples (high support) compared to the total dataset size (n). The exponential function is used to rapidly amplify values as the input grows’.

1. Performance per label: all these metrics were calculated for each label individually.

Where TP, TN, FP, FN are the number of True Positives, True Negatives, False Positives and False Negatives, respectively.

At the time of experimentation, the Tensorflow library did not offer the HParams utility, which provides hyperparameter tuning facility. Therefore, the overall model or the parameters used were chosen manually via the process of hit and trial. We specifically tried multiple values of learning rate and batch size. However, the specifics of each trial were not formally recorded.

**Retraining**

A background service was set up for retraining the model each night, which appended all new examples in the Mongo DB database to the base data set after pre-processing, irrespective of whether any new data was added or not. The snapshot of this retrained model was stored in the database with timestamp.

The newly added examples were pre-processed via a predefined process.

To ensure that the production environment was always using the best model available, the newly trained model was compared with the current model. The new model was uploaded to production only if it outperformed the prior model in Trust score. The Program team monitored all historical models on a dashboard.

The full architecture of the model is represented below:

The model’s infrastructure is logically divided into two components, namely the Environment and the Application. The Environment contains the processes specific to extracting raw data from the EPI database and applying standard operations to train the model. All of the sub-processes in this component are contained in a separate server, which periodically executes a job to retrain a new prediction model and replace it with the previous version in case the new model shows better performance and accuracy. Tensorflow, TFLearn and NLTK are different libraries which are used to train the prediction model.

The Application is responsible for the interfacing and API integrations. Inside the Application component is a web application built on Django (a Python-based framework) which includes a custom built API to interact with the Environment, the EPI registry, as well as external gateways for messaging. The Application component is responsible for handling the incoming queries and categorizing them for sending to the bot or the human responder.

Supplementary Figure 1
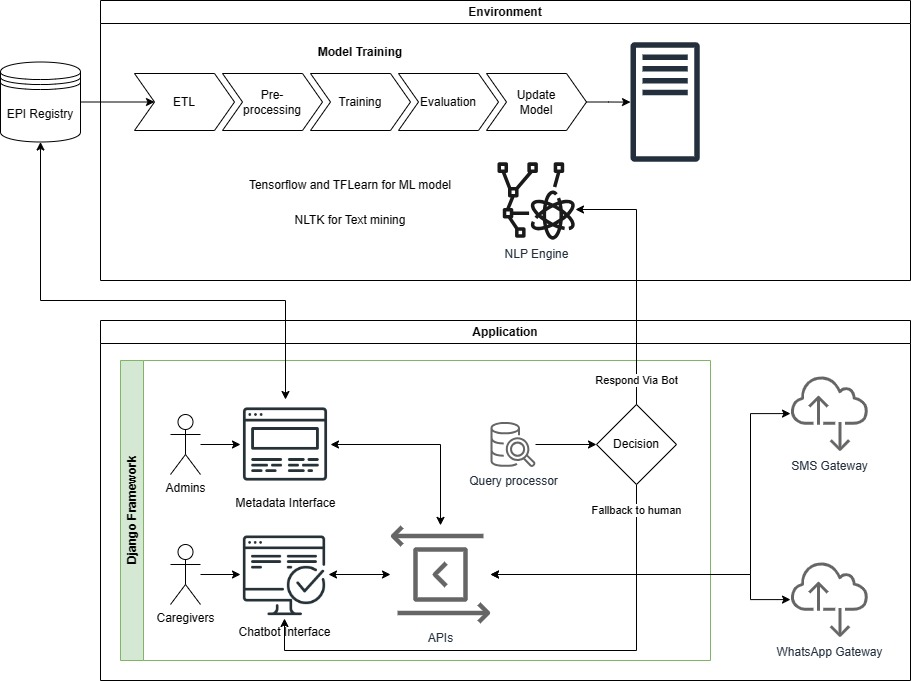


1. Abadi, Martín, Paul Barham, Jianmin Chen, Zhifeng Chen, Andy Davis, Jeffrey Dean, Matthieu Devin et al. "Tensorflow: a system for large-scale machine learning." In *Osdi*, vol. 16, no. 2016, pp. 265-283. 2016. [↑](#footnote-ref-1)
2. Pakistan’s regional format is DD/MM/YYYY, however DD-MM-YY is also commonly used. [↑](#footnote-ref-2)
3. Natural Language Tool Kit is a widely used library for common text mining functions. [↑](#footnote-ref-3)
4. Goodfellow, I., Bengio, Y. and Courville, A., 2018. Softmax units for multinoulli output distributions. Deep Learning. [↑](#footnote-ref-4)
5. Kingma, D.P. and Ba, J., 2014. Adam: A method for stochastic optimization. *arXiv preprint arXiv:1412.6980*. [↑](#footnote-ref-5)
